# Insights into Mechanisms of Pheochromocytomas and Paragangliomas Driven by Known or New Genetic Drivers

**DOI:** 10.3390/cancers13184602

**Published:** 2021-09-14

**Authors:** Shahida K. Flores, Cynthia M. Estrada-Zuniga, Keerthi Thallapureddy, Gustavo Armaiz-Peña, Patricia L. M. Dahia

**Affiliations:** 1 Department of Medicine, University of Texas Health San Antonio, San Antonio, TX 78229, USA; shahida.flores@gmail.com (S.K.F.); estradazunig@uthscsa.edu (C.M.E.-Z.); thallapuredd@livemail.uthscsa.edu (K.T.); armaizpena@uthscsa.edu (G.A.-P.); 2 Mays Cancer Center, University of Texas Health San Antonio, San Antonio, TX 78229, USA

**Keywords:** pheochromocytomas, paragangliomas, mutations, susceptibility genes, driver mutations, hereditary, germline, somatic, environment, variants, tumor suppressor genes, metastatic, treatment, RNAseq, next generation sequencing

## Abstract

**Simple Summary:**

Pheochromocytomas and paragangliomas are rare neuroendocrine tumors that are often hereditary. Although research has advanced considerably, significant gaps still persist in understanding risk factors, predicting metastatic potential and treating aggressive tumors. The study of rare mutations can provide new insights into how pheochromocytomas and paragangliomas develop. In this review, we provide examples of such rare events and how they can inform our understanding of the spectrum of mutations that can lead to these tumors and improve our ability to provide a genetic diagnosis.

**Abstract:**

Pheochromocytomas and paragangliomas are rare tumors of neural crest origin. Their remarkable genetic diversity and high heritability have enabled discoveries of bona fide cancer driver genes with an impact on diagnosis and clinical management and have consistently shed light on new paradigms in cancer. In this review, we explore unique mechanisms of pheochromocytoma and paraganglioma initiation and management by drawing from recent examples involving rare mutations of hypoxia-related genes *VHL, EPAS1* and *SDHB*, and of a poorly known susceptibility gene, *TMEM127.* These models expand our ability to predict variant pathogenicity, inform new functional domains, recognize environmental-gene connections, and highlight persistent therapeutic challenges for tumors with aggressive behavior.

## 1. Overview and Current Status of Genetic Drivers

Pheochromocytomas and paragangliomas (PPGLs) are rare neural crest derived tumors with an incidence of 500 to 1600 cases per year [1,2]. Pheochromocytomas arise from adrenomedullary chromaffin cells and paragangliomas arise from extra-adrenal chromaffin cells of the sympathetic paravertebral ganglia of thorax, abdomen, and pelvis or chief cells that form the paraganglia of glossopharyngeal and vagal nerves in the neck and base of the skull [3]. While pheochromocytomas and thoracic-abdominal paragangliomas often produce catecholamines, head and neck paragangliomas are almost invariably non-secreting [4]. PPGLs are predominantly benign, and malignancy is only established by the detection of metastasis, which occurs in approximately 30% of paragangliomas and 10–15% of pheochromocytomas. Currently there are limited options for treatment of metastatic PPGLs [5,6].

**Figure 1:**
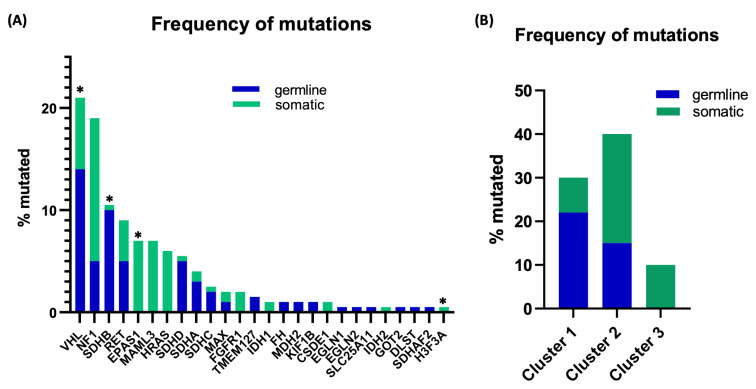
Approximate mutation frequency of genes implicated in PPGLs with a known genetic driver. Data were compiled from published series [10,14,16,17,18] and our own cohort regardless of age groups and may reflect referral bias. (**A**) Mutation distribution based on individual genes and (**B**) cluster type. Tumors with unknown genetic drivers are not shown. For the purpose of this representation, mutation frequencies of uncommon genes have been depicted as 0.5%. * genes that can be post-zygotically mutated. The genes implicated in PPGLs, with various degrees of supporting evidence are: *NF1, VHL, RET, SDHA, SDHB, SDHC, SDHD, SDHAF2, TMEM127, MAX, EPAS1, HRAS, FH, EGLN1, EGLN2, MDH2, FGFR1, CSDE1, MAML3, GOT2, SLC25A11, H3F3A, DLST, IDH1, IDH2, KIF1B, MET*.

PPGLs are remarkable for their high heritability rate and genetic diversity. More than 20 genes have been implicated in PPGL [7,8,9]. Mutations of these genes occur in a mutually exclusive manner through germline (~30–40%) or somatic (30%) transmission (Figure 1A) [10,11]. Within the domain of hereditary mutations, genes that predispose to genetic syndromes include *RET* (multiple endocrine neoplasia type 2A and 2B), *VHL* (von Hippel Lindau disease), *NF1* (neurofibromatosis type 1) and *SDH* subunit genes (hereditary paraganglioma syndromes types 1–5) [12]. *TMEM127, MAX, FH* and *MDH2* genes have also been linked to germline mutations [13]. However, *NF1*, *VHL, RET,* and *MAX* can also be somatically mutated. Genes exclusively associated with somatic mutations include *EPAS1, ATRX*, and *HRAS.* [10,14,15,16,17]. Besides germline and somatic mutations, mosaicism (post-zygotic mutation) has been reported in *EPAS1, H3F3A*, *VHL* and *SDHB*, and has been historically associated with *NF1*, although not specifically in the context of PPGLs [16,18]. Recently, gene fusions have been recognized in PPGLs, especially those involving the MAML3 transcription factor, including the *UBTF-MAML3* fusion [14]. Other genes (listed in Figure 1A) have only been reported in a few cases, and the evidence supporting their direct role in PPGLs still remains limited [19].

PPGLs have been classified into three clusters according to the molecular pathways involved in their pathogenesis [14]. Cluster 1 consists of the pseudohypoxia pathway and includes tumors with either germline or somatic mutations in *VHL, SDHA/B/C/D/AF2, EPAS1, EGLN1, EGLN2, FH, SLC25A11,* and *MDH2* (Figure 1B). This cluster is also subdivided into genes associated with the tricarboxylic acid (TCA) or mitochondrial function (*SDH, FH, MDH2, SLC25A11, IDH1/2*), also referred to as C1A group, and other hypoxia pathway-related genes, or C1B (*VHL, EPAS1, EGLN1/2* genes) [20]. Cluster 2 is characterized by kinase signaling and protein translation pathways and includes PPGLs with germline or somatic mutations of *RET, NF1, TMEM127, HRAS, FGFR1,* and *MAX*. Cluster 3 has been recognized more recently, is related to activation of targets of the *WNT1* transcription factor and includes *MAML3* fusion genes and truncating mutations in *CSDE1* [14,21]. This classification underlies the diverse mechanisms and signals that can initiate PPGLs, although it remains challenging to predict the disease course [22]. Although the biological behavior of PPGLs cannot be anticipated, specific genotypes have been associated with an increased risk of metastasis. For example, *SDHB* mutations confer a higher risk of metastatic progression. Similarly, somatic *MAML3* fusions, often accompanied by disruption of *TERT* and/or *ATRX* mutations are enriched in aggressive and/or metastatic tumors [5,23,24].

## 2. Leveraging Clinical and Genetic Data for Classification and Patient Management

Current evidence supports genetic testing as a key component of the management of patients with PPGL to guide treatment selection and follow-up surveillance [1,25]. Disease presentation and the likelihood of identifying a causative germline mutation will vary depending on the molecular class of the PPGL [26]. For example, tumors belonging to Cluster 1 (pseudohypoxia) may present as either pheochromocytoma or paraganglioma, often occur at a younger age (especially those with germline *VHL* mutation) and frequently manifest as multiple and/or recurrent. Metastatic disease, especially if *SDHB* related, is enriched in this group [27]. These tumors are characteristically deficient for the enzyme which converts norepinephrine (NE) to epinephrine (Epi), phenyl-ethanolamine N-methyltransferase (PMNT). For these reasons these PPGLs are strictly noradrenergic and can be diagnosed preferentially by elevated NE levels [26,28]. A germline mutation can be detected in most cases of C1A-related PPGLs, while the rate of germline mutation is lower in C1B cases [26]. In contrast, around 20% of Cluster 2 cases (kinase signaling group) are associated with a germline mutation. These patients have a broad age of presentation that can be modulated by the specific gene mutated, usually peaks between 40–50 years of age and present as benign pheochromocytomas [26,29]. Not infrequently these tumors are multiple, especially related to MEN 2A/2B, but to a lesser extent *TMEM127*- and *MAX*-mutant cases. Cluster 3 (Wnt-altered) presents as pheochromocytomas that are often metastatic or recurrent, although studies are still limited to few cases. There have not been germline variants associated with this cluster to date. Both Cluster 2 and 3 express PMNT and are associated with elevated Epi/NE levels [14,30].

PPGL localization and possible metastasis identification usually involve computed tomography (CT) or magnetic resonance imaging (MRI) as the initial step, regardless of genotype. However, in suspected metastatic cases, recurrent disease, or if radionuclide-based therapy is being considered, distinct functional imaging studies can be utilized, such as ^123^I-metaiodobenzylguanidine (MIBG), 6-^18^F-fluoro-L-dopa (^18^F-FDOPA), ^18^F-fluorodeoxyglucose (^18^F-FDG), and gallium-68 DOTATATE (^68^Ga-DOTATATE). Once again, molecular knowledge can influence the functional imaging choice. For example, Cluster 2-type PPGLs have high avidity for ^18^F-FDOPA but a low-to-moderate ^18^F-FDG uptake [26]. In contrast, cluster 1-related PPGLs with *VHL* or *EPAS1* mutations display high uptake of ^18^F-FDOPA and ^18^F-FDG [26,31]. Genotype-functional imaging associations are more complex in *SDH*-related PPGLs. In these tumors, nuclear imaging studies will depend mainly on the tissue of origin, with ^18^F-FDOPA being characteristically positive for head and neck PGLs but not for sympathetic PPGLs [31]. Also, *SDH*-related tumors, particularly *SDHB* mutants, are known to show poor sensitivity to ^123/131^I-MIBG compared to other radiopharmaceuticals, like ^18^F-FDG PET/CT and ^68^Ga-DOTATATE [32,33]. Furthermore, ^68^Ga-DOTATATE demonstrates superiority to other available functional studies regardless of location, if *SDH*-related parasympathetic PGL or metastatic PPGL is identified [34].

**Figure 2:**
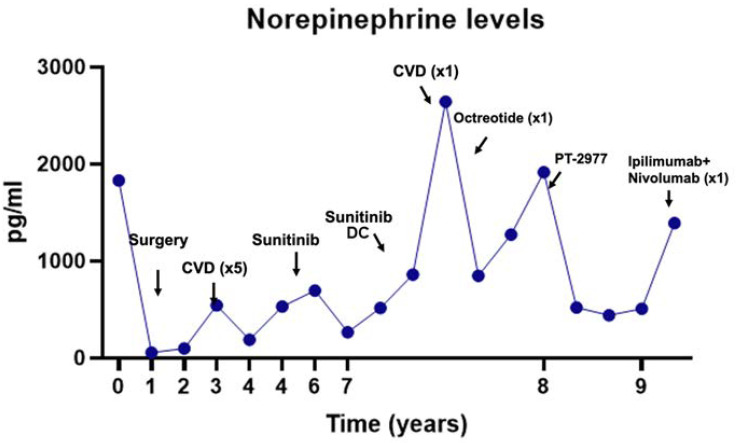
Plasma norepinephrine (NE) levels of a patient with a pathogenic germline *SDHB* mutation diagnosed with a retroperitoneal paraganglioma, who progressed with metastases and underwent multiple lines of treatment over the course of her disease. NE levels are tracked closely with the tumor burden and symptoms. Bone metastases were detected two years post-surgery. The patient received CVD followed by sunitinib, with the initial control of disease, however, both therapies were eventually discontinued (DC) due to adverse side effects. After disease progression, new attempts were made with sunitinib and CVD, although once again drugs were poorly tolerated. One dose of octreotide depot was given to attempt symptomatic control of the disease. Next, the patient was enrolled in the Phase 1 clinical trial for a HIF2α inhibitor (PT-2977/ MK6484, NCT02974738). The patient had clinical, biochemical, cellular, and molecular responses mainly demonstrated by a decrease in NE, development of anemia, a common on-target effect of HIF2 inhibition, and reduced expression of HIF2α target genes (not shown) and remained stable for 8 months. Despite this improvement, the disease progressed, and the HIF2α inhibitor was discontinued. The patient initiated a trial with CTLA-4 and PD-1 inhibitors (NCT02834013) but only tolerated one cycle. Disease progressed rapidly and the patient died a few months later. This case illustrates two critical timepoints during disease evolution that remain gaps in the field: determining the basis for the rapid increase in disease burden and emergence of resistance to targeted therapy could inform treatment choices in patients with metastatic pheochromocytoma and/or paraganglioma.

The first line of treatment for all PPGLs should be tumor resection with pre-operative management of catecholamine-related symptoms that are usually achieved by alpha-blockade, regardless of mutation status [1,35]. However, knowledge of the genotype impacts on surgical planning, as patients diagnosed with, or at risk of, bilateral pheochromocytomas are recommended to undergo cortical-sparing surgery [36]. However, not all PPGLs are amenable for surgery due to metastatic disease, surgically challenging tumor location, or extensive recurrence. In cases where surgery is not feasible, tumor burden, disease progression, or symptomatic status should guide treatment options that include local therapies (radiotherapy, radiofrequency ablation, embolization, among others), radionucleotide therapy and chemotherapy [5]. Radionucleotide therapy with ^131^MIBG can be considered when ^123^MIBG diagnostic scans demonstrate avid uptake. A recently FDA-approved, high-specific activity version of ^131^MIBG showed a response in more than 90% of patients, with tumor reduction in 25% of cases [37], although long-term follow-up of this drug is still lacking. Cytotoxic radionuclide therapy with ^177^Lu-DOTATATE shows promise as a therapeutic option that provides less toxicity than ^131^MIBG; however, although studies are still limited [38]. Systemic chemotherapy with cyclophosphamide, vincristine, doxorubicin (CVD) can reduce tumor burden, decrease catecholamines, and improve blood pressure in only 30–40% of patients, and data with other agents, such as temozolomide are limited to small cohorts [5]. Given this limited effectiveness of systemic therapies, targeted therapies have been tested, though usually outside of clinical trials [5,39]. The highly vascular nature of PPGLs, and increased VEGF expression and activity especially notable in cluster 1 tumors justifies the use of tyrosine kinase inhibitors (TKI) with antiangiogenic properties, such as sunitinib, pazopanib, axitinib, cabozantinib, lenvantinib, and dovitinib [40,41]. Another potential and even more promising therapy targeting molecular disruption of PPGLs involve HIF inhibitors, in particular HIF-2α, which has been identified as one of the main oncogenic drivers in PPGL development and is overexpressed in *VHL*, *SDH,* and *EPAS1*-mutant PPGLs [42,43]. A novel class of HIF-2α-specific inhibitors showed promising results in advanced clear cell-renal cell carcinoma (cc-RCC) [44]. This drug, belzutifan (previously known as PT2977), received FDA approval in August 2021 for the treatment of VHL-related cc-RCC, hemangioblastomas and pancreatic neuroendocrine tumors (https://www.fda.gov/drugs/resources-information-approved-drugs/fda-approves-belzutifan-cancers-associated-von-hippel-lindau-disease, accessed on 27 August 2021). This is an important milestone that will accelerate the development of new trials [42,43], including advanced and/or metastatic PPGLs (NCT04924075). Immunotherapy is another area of interest in the treatment of cluster 1 related PPGLs, as pseudohypoxia may prevent immune recognition of the tumors via mechanisms involving increased expression of the immune checkpoint protein programmed death-ligand 1 (PD-L1) and inactivating cytotoxic T cell lymphocytes. Pembrolizumab, nivolumab and ipilimumab are being studied as possible therapeutic options [45]. Figure 2 illustrates the challenges of treating patients with metastatic PPGL and the need for additional research to better understand the events underlying rapid disease progression after months or years of indolent metastatic growth, and molecular determinants of acquired resistance to targeted therapy. Advances on these fronts will be critical to refine treatment strategies and improve patient outcomes.

## 3. Detecting and Interpreting Variants: Protocols and Challenges

Patients with PPGLs should be engaged in genetic testing [1,35]. The relevance of genetic diagnosis is demonstrated by its positive impact on patient outcomes [46]. Next-generation sequencing (NGS) technology has emerged as a valuable tool capable of simultaneously evaluating multiple susceptibility genes in the same assay [47]. This methodology significantly improves the performance of PPGLs genetic testing compared with conventional methods, increasing the rate of variant identification [10,16]. At the same time, this approach leads to the detection of rare and novel variants, and the task of defining their pathogenicity becomes a relevant challenge [47].

According to the ACMG Standards and Guidelines [48], several lines of evidence are needed to support the classification of a variant as pathogenic or likely pathogenic. Variant classification requires careful interpretation of a combination of information including (a) the type of variant, (b) the frequency of the variant, (c) the occurrence of the variant in clinically-related databases, (d) literature citations of the variant, (e) functional evaluation of the variant, (f) in silico predictions of variant effect, (g) analysis of co-segregation of disease in the family, (h) concordance with phenotype, and (i) co-occurrence of pathogenic variants [47]. The latter is an increasingly likely scenario observed in NGS-based studies, which adds to the complexity of interpreting variant relevance [49]; however, this subject will not be discussed in this brief review. Not uncommonly, very strong evidence (a null variant in a gene where the loss of function is an established disease mechanism) and strong evidence (functional studies support a damaging effect; higher prevalence of variant in affected individuals vs. controls, etc.) that would support pathogenicity is not available. This is especially true for rare, genetically heterogeneous diseases, such as PPGLs which can arise due to a germline, somatic or mosaic variant in one of many susceptibility genes. As a result, a substantial number of variants, especially missense substitutions, identified in PPGL susceptibility genes are currently classified as variants of uncertain significance (VUS) pending additional support for pathogenicity [10,16]. Functional studies are recommended to assess the pathogenicity of variants, which may be resource-intensive [18,22,47].

## 4. A Workflow to Identify a Driver Mutation in PPGLs

**Figure 3:**
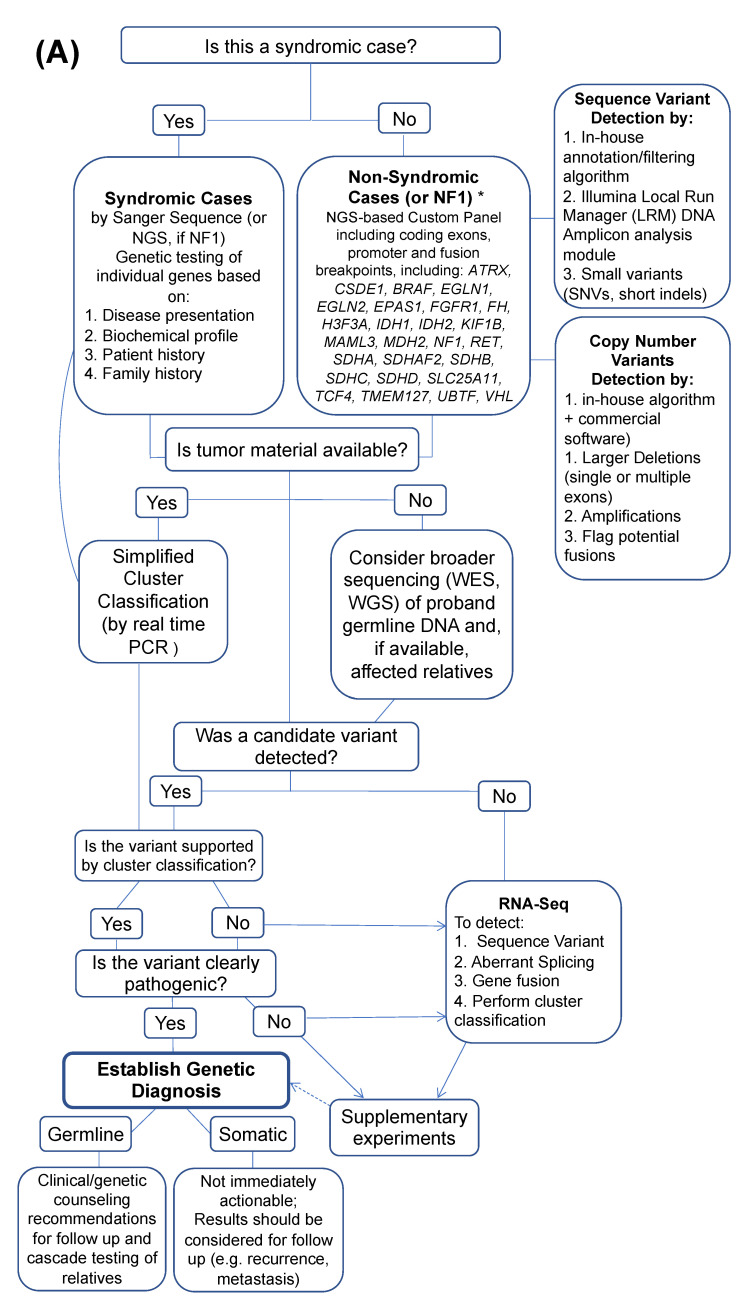

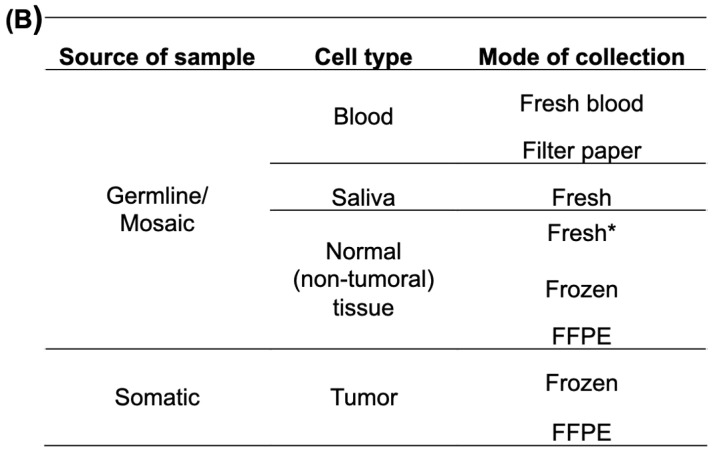
(**A**) A proposed workflow to identify driver mutations in pheochromocytoma and/or paragangliomas (PPGLs). The process is modified based on the type of sample available for analysis and initial clinical information. The ultimate goal is to establish a germline or somatic genetic diagnosis. In some cases, extensive experimentation may be necessary, as shown by directional lines. A definitive, unambiguous diagnosis may not be achieved in all cases (dashed line), and additional research is required. Limitations may include samples with only germline material available, without family history/or samples from informative relatives, and no clear candidate variant. * areas with lower coverage are supplemented by Sanger sequencing; NGS = next-generation sequencing; WES = whole exome sequencing; WGS = whole genome sequencing (**B**) Materials used for analysis; FFPE = formalin-fixed, paraffin-embedded; * cell culture compatible media.

Multiple strategies have been employed for the genetic diagnosis of PPGLs. Our group adopted a flexible workflow (NCT03160274) depicted in Figure 3A. This process involves parallel testing of blood and tumor tissue, either fresh frozen or as formalin-fixed, paraffin-embedded material (Figure 3B). While this protocol includes both germline and tumor samples for DNA-based screening whenever possible, it prioritizes tumor tissue processing. This approach allows for improved data interpretation [50], by enabling the detection of somatic events or suspected areas of copy number variation, including loss of heterozygosity.

For clearly syndromic cases, the first step of this workflow may include targeted testing. For non-syndromic PPGLs, a next-generation sequencing (NGS)-based custom panel of 28 genes is used (Figure 3A). Libraries are processed and sequenced at high depth (>500× average) in an Illumina MiSeq instrument, easily scalable to higher capacity instruments for higher throughput, as needed (e.g., Illumina NextSeq). Data are analyzed for sequence variants of interest and copy number changes, or, if tumor tissue is available, suggestive systematic gain/loss patterns of known fusion partner genes. If high-quality tumor RNA is available, sequencing is followed by a focused transcription profiling step based on real-time PCR of tumor cDNA. This step has two purposes: (i) to determine whether the expression pattern of tumors with a detected candidate driver mutation matches the expected cluster group, and (ii) to guide the subsequent investigation of mechanisms that drive pathogenicity in tumors with suspected VUS or those with an unknown driver event based on cluster membership. The genes included in this focused classification were modeled on top classifiers of the three main expression clusters previously reported [14,21,51,52] and other curated expression data.

Tumor samples without an identifiable variant are subjected to whole transcriptome sequencing (RNAseq). This approach can provide multiple levels of information to improve driver gene detection [53,54,55]. Although comprehensive analysis of whole transcriptome data requires bioinformatics expertise, the broad use of RNAseq-based algorithms has simplified this process [53,56]. First, it provides sequence data of the whole transcriptome, beyond the known PPGL genes, enabling the identification of potentially novel candidate driver genes. Although the depth of coverage of conventional RNAseq data tends to be generally lower than that provided by typical custom DNA panels, and can be subject to variability dependent on transcription instability of certain mutants, high-depth RNAseq can improve detectability [57]. Secondly, the data can also reveal genes targeted by aberrant splicing that may explain atypical and/or suspect variants. Thirdly, RNAseq data also generates expression classifications that can help support putative candidate variants (e.g., pseudohypoxia expression signature of a sample with a *SDHB* VUS). A fourth advantage of RNAseq is its ability to predict putative in-frame gene fusions that may have an oncogenic role in PPGLs. Putative fusions can be orthogonally verified by designing specific breakpoint spanning primers and by sequencing independent tumor samples with a shared expression profile. Thus, the incorporation of tumor RNAseq for fusion and splicing aberration detection can expand the characterization of novel structural variants. When integrated with expression profiles, these data can also provide insights into the potential dominant signaling disruption (e.g., pseudohypoxia).

Additional steps of the workflow are guided by individual findings [47]. For example, these analyses can be complemented by immunohistochemical staining of selected, well-established targets [58], or novel targets, to help support cluster membership and the functional impact of candidate variants. Additional functional experiments are usually tailored to the candidate gene and variant type, as exemplified in the next section. Other analytical platforms also contribute to improved diagnosis and classification, when integrated with sequencing, transcription and immunohistochemical analyses. Epigenetic (especially DNA methylation profiling, but also analysis of posttranslational modifications) and metabolite profiling can help to narrow down the classes of possible susceptibility gene mutations, as well as potential consequences of candidate variants [19,50,59,60].

## 5. Lessons Learned from Atypical/Novel/Unsuspected Genetic Disruptions

This session addresses the relevance of exploring rarer or atypical variants and how these investigations can reveal driver mutations and mechanisms of PPGL tumorigenesis, illustrated with examples from our cohort. Some PPGL susceptibility genes, like the transmembrane protein encoding gene *TMEM127*, are poorly known upon their identification [61]. TMEM127 has been previously described as a tumor suppressor, an endomembrane protein, and a negative regulator of mTOR signaling [61,62]. Tumor suppressor genes are often inactivated by frameshift and nonsense/truncating variants, but the effects of missense variants are more difficult to characterize. Individually, missense variants observed in PPGL patients and families may not reveal much information, but collectively, they can highlight specific functional protein hotspots. Recently we used in vitro transient expression of cDNA constructs to investigate a cluster of missense *TMEM127* variants that suggested the presence of a putative functional domain not previously described in the N-terminal region [63]. We had reported earlier that membrane binding ability appears to be required for TMEM127 function [64,65], therefore analysis of variant subcellular localization patterns served as an efficient first-pass approach in evaluating loss of structure/function. All missense variants in the N-terminal cluster had a diffuse/cytoplasmic pattern, in contrast with the punctate, endomembrane pattern of wild-type (WT) TMEM127 indicating that these variants lost their membrane binding ability. Moreover, these variants were rapidly degraded, in favor of a loss of function defect. Through this process and additional topology studies, a fourth TMEM127 transmembrane domain was identified [63]. These findings were recently supported by highly accurate deep learning protein structure predictions [66]. At the same time, distinctive variants can also reveal key protein features. A C-terminal, frameshift variant, disrupting the region downstream of the last transmembrane domain of *TMEM127*, was found to display a unique, plasma membrane bound pattern. This observation suggested that the variant lost its internalization capability. Further analysis revealed that an atypical endocytic signaling motif resided in the C-terminal tail and was necessary for effective localization of TMEM127 [63].

Another example of atypical variants with unsuspected functional consequences is illustrated by synonymous variants. Unless they are located close to exon-intron boundaries, where they could disrupt donor and acceptor splice sites, synonymous variants are often filtered out during screening because they are not predicted to result in a change to the protein sequence. However, considerations need to be made that synonymous variants occurring in the middle of an exon may also have an effect on splicing, as demonstrated recently with *VHL* [67,68]. Although the mechanism is not understood, a synonymous variant in the middle of exon 2 of *VHL* at proline 138 (c.414A > G, p.138=) results in a splicing effect that omits exon 2 from the transcript, resulting in an in-frame transcript consisting of exon 1 and exon 3 [67,68]. Importantly, exon 2 encodes most of the oxygen-dependent degradation domain (ODD) of VHL, the HIF binding site, and its absence leads to reduced HIF2α degradation, similar to other loss-of-function *VHL* mutations. Several families carrying this variant have now been reported, enabling reclassification of this variant as pathogenic [67,68].

The reports above demonstrate the utility of functional studies in expanding and redefining our knowledge of existing genes as well as supporting variant classification. Over time and with long-term follow up these observations may be updated to uncover new genotype-phenotype associations of value in implementing clinical surveillance practices. Despite these efforts, there remain tumors with undefined driver events. These tumors may carry disruptions of the noncoding genome, epigenetic events, or involvement of multiple genes, and their study will require additional approaches [69].

## 6. Epistatic Interactions between Genetics and the Environment

Establishing causality of gene-environment interactions in cancer, defined by co-participation in the same causal mechanism, is challenging [70]. Several disease models have emerged in which cancer development has been traced to specific types of environmental stress [70]. However, the ability to precisely measure the impact of exposure to environmental stressors, such as radiation, toxins, or oxygen variability, and define their direct role in the acquisition of genetic mutations which can influence disease risk and severity is limited.

An intriguing natural model of environmental risk is represented by patients with cyanotic congenital heart disease (CCHD), a group of diseases caused by complex heart defects present at birth that result in low blood oxygen levels (hypoxemia) [71]. Even after corrective surgeries, some degree of hypoxemia may remain, which creates a state of chronic systemic hypoxia in these patients [72]. It has long been recognized that CCHD patients have a higher incidence (~5-fold) and earlier occurrence of PPGLs compared to the general population [72,73,74]. Although a molecular basis had not been previously established, it was considered that these two diseases might share an inherited genetic susceptibility. However, recent studies support that the development of PPGLs in CCHD patients is linked to a somatic event in one of the PPGL susceptibility genes. Specifically, the *EPAS1* gene, which codes for the hypoxia inducible factor HIF2α, which plays a significant role in the hypoxia-response pathway, was found to be susceptible to somatic mutations at critical residues [75]. In a study of six tumor samples from five CCHD patients, including five sympathetic PPGLs and one carotid body paraganglioma (CB-PGL), we found that four out of five sympathetic PPGLs displayed a somatic *EPAS1* mutation affecting either alanine 530 or proline 531 [75]. As these residues play a key role in regulating HIF2α stability, the resulting amino acid changes prevent degradation and, hence, confer a constitutively active status for HIF2α [76]. Notably, these patients had no germline mutations of known PPGL susceptibility genes, supporting a driver role for the somatic *EPAS1* mutations [75]. Interestingly, the PGL of the single patient without a somatic *EPAS1* mutation showed an SDHA/SDHB immunohistochemistry pattern compatible with deficient SDH function, suggesting a qualitatively distinct mechanism of tumorigenesis in this case. The single carotid body PGL in this series, which arose in the same patient with an *EPAS1* mutant pheochromocytoma, also did not carry a somatic *EPAS1* mutation.

In the sections above, we emphasized the overrepresentation of hypoxia-related genes in mutated PPGLs (*VHL, SDH* subunits, *EGLN1/2, FH, IDH, EPAS1*), highlighting the relevance of this pathway for tumor development [42]. While most PPGLs within the pseudohypoxia cluster result from germline variants, suggesting an early event, the *EPAS1* gene is targeted instead by somatic mutations [14,77]. These somatic *EPAS1* mutations are detected at a frequency no higher than 7% in cohorts of generic PPGLs [14,77]. In contrast, in PPGLs arising in patients with CCHD the frequency of *EPAS1* mutations is markedly elevated, at 80% [75]. The timing of the emergence of the *EPAS1* mutation within the PPGL tumorigenesis process in CCHD patients remains unknown. However, the specific conditions experienced by these patients, which include prolonged tissue exposure to low circulating oxygen levels, may act as an environmental cue that favors PPGL development through somatic mutations that selectively target a key component of the hypoxia response. These observations suggest that sympathetic cells of the adrenal and paraganglia are uniquely sensitive to the CCHD environment, similar to other cell types that experience specific genetic vulnerabilities in the presence of particular external factors, much like targeted therapy-induced resistant mutations in cancer [78].

At the same time, CB-PGLs differ from other PPGLs in the cell of origin (chief cells instead of chromaffin cells), and, hence, may have genetic vulnerabilities distinct from chromaffin-derived PPGLs [79]. Of note, individuals living in certain high-altitude areas, such as the Andes, who are exposed to low relative ambient oxygen pressure have a higher incidence of CB-PGLs [73,74]. In some cases, CB-PGL development has been attributed to germline variants in *SDHB* [80] or *SDHD* [81]. However, not all tumors have detectable variants in SDH genes [82]. Future studies will be needed to determine whether chief cells have a distinct vulnerability to mutations and whether other genes related to the hypoxia response can also be implicated in these tumors. Regardless, the remarkable association between CCHD and *EPAS1* mutated-PPGLs should spur studies to further investigate and model the impact of environmental influences in PPGL tumorigenesis that may also illuminate our knowledge of other cancers.

## 7. Conclusions

Great advances have been achieved in the knowledge of the genetic basis of PPGLs. However, the driver event remains unidentified in at least one-third of the cases. Importantly, the ability to recognize molecular identifiers of metastatic risk persists as an unattained goal. Bridging these gaps will require optimization of workflows for genetic diagnosis, improvement of variant annotation and the recognition of atypical genetic disruptions that shed light on novel disease mechanisms. Overcoming these challenges will require unified efforts of researchers in this field.
